# Application of tDCS in children with cerebral palsy: A mini review

**DOI:** 10.3389/fped.2022.966650

**Published:** 2022-09-20

**Authors:** Lin Tang, Yuwei Wu, Jiayin Ma, Yan Lu, Ling Wang, Chunlei Shan

**Affiliations:** ^1^Department of Rehabilitation Medicine, Yueyang Hospital of Integrated Traditional Chinese and Western Medicine, Shanghai University of Traditional Chinese Medicine, Shanghai, China; ^2^Department of Rehabilitation Medicine, Seventh People's Hospital of Shanghai University of Traditional Chinese Medicine, Shanghai, China; ^3^Engineering Research Center of Traditional Chinese Medicine Intelligent Rehabilitation, Ministry of Education, Shanghai, China; ^4^School of Rehabilitation Science, Shanghai University of Traditional Chinese Medicine, Shanghai, China

**Keywords:** cerebral palsy, tDCS, motor function, cognitive and language functions, prospect

## Abstract

Cerebral palsy (CP) refers to a group of diseases characterized by persistent central dyskinesia, postural development disorder and activity limitation syndromes caused by nonprogressive brain injury in the developing fetus or infant, which is often accompanied by sensory, cognitive and attention disorders. The routine rehabilitation methods for children with CP mainly include physical therapy, occupational therapy, speech therapy and other methods. In recent years, noninvasive brain stimulation (NIBS), as a relatively new intervention method, has been widely used because of its potential to regulate cortical excitability and plasticity. Transcranial direct current stimulation (tDCS) is an NIBS technique that is easier and more convenient to perform. It does not require patients to remain stationary for a long time or have a significant impact on treatment results due to children's frequent activities. Compared with other NIBS techniques, tDCS has greater flexibility and no strict restrictions on patients' activities; it also helps the therapist conduct occupational therapy or speech therapy while a child receives tDCS, which markedly reduces the treatment time and avoids burnout due to a long treatment duration. Thus, tDCS is a better and more convenient intervention for CP children and warrants further exploration. Accordingly, this article reviews tDCS application in children with CP and discusses tDCS application prospects for such children to promote its expansion in clinical practice.

## Introduction

Cerebral palsy (CP) refers to a group of diseases characterized by persistent central motor, postural developmental disorders and activity limitation syndromes caused by nonprogressive damage to the developing fetal or infant brain, which is often accompanied by sensory impairment, cognitive impairment, attention disability, sleep disorder and other symptoms (1–3). Epidemiological survey results show that the prevalence of cerebral palsy between the ages of 0 and 18 years in different regions of China is ~2.07%, and that the comorbidity rate of cerebral palsy and other diseases increased rapidly during 2008 and 2019 (4). Most CP children have motor dysfunction and speech dysfunction, and independent movement ability affects the psychological condition of these children. Family support and social support needs are also higher, which imposes a financial burden and pressure on the family (5).

At present, the conventional rehabilitation methods for CP children mainly include physical therapy, occupational therapy, speech therapy and others. Recently, noninvasive brain stimulation (NIBS), as a relatively new intervention, has been widely used due to its potential to modulate cortical excitability and plasticity, and its mechanism is speculated to involve certain excitation or inhibition effects on various brain regions (6).

Among many NIBS techniques, transcranial magnetic stimulation (TMS) and transcranial direct current stimulation (tDCS) are two of the more commonly used techniques, and tDCS is a simpler and more convenient intervention for children. It does not require patients to remain still for extended periods or significantly affect treatment outcomes due to children's frequent activities (7).

tDCS is a noninvasive technique that utilizes constant, low-intensity direct current (usually 1–2 mA) to modulate neuronal activity in the cerebral cortex. It mainly acts on the cerebral cortex with weakly polarized direct current through two electrodes placed on the scalp, and the current modulates neuronal excitability by changing the resting membrane potential of neurons (8). The excitatory or inhibitory effects of tDCS on cortical activity are thought to depend on electrode polarity, with the resting membranes of neurons near the cathode becoming progressively hyperpolarized and less excitable, while the membranes of neurons near the anode are slightly depolarized, thus becoming more excitable (6).

Compared with TMS, tDCS has greater flexibility and no strict restrictions on patients' activities. It also helps therapists perform occupational therapy or speech therapy while a child receives tDCS, which markedly reduces the treatment time and avoids burnout due to a long treatment duration.

tDCS seems to be a better and more convenient intervention method for children with cerebral palsy and warrants further exploration. Therefore, this article reviews tDCS application in children with CP and discusses tDCS application prospects for such children to promote its expansion in clinical practice.

## tDCS improves motor function in CP children

Several common motor disorders affect CP children, such as abnormal muscle tone, poor hand function, decreased muscle strength, abnormal gait patterns and balance dysfunction (9). Due to the difference between adults and children, it is indeed unclear whether the theory of improving motor function by affecting the connectivity of the motor network between cerebral hemispheres is applicable to the developing brain of children (10). However, some researchers support the existence of inhibitory imbalance between the hemispheres in children with unilateral cerebral palsy (11, 12), which holds that an inhibitory imbalance exists between the hemispheres in the M1 area (primary motor cortex) on the injured side and the M1 area on the noninjured side, leading to a decrease in the activity of brain regions on the injured side and an increase on the noninjured side. Therefore, in most tDCS applications in CP children, the anode is applied to the M1 area on the injured side to increase excitability, or the cathode is applied to the M1 area on the noninjured side to reduce excitability.

### Upper limb function

Reports of tDCS improving upper limb motor function in CP children are relatively limited. Three clinical studies have combined tDCS with other interventions, demonstrating that tDCS combined with other interventions can improve upper limb motor function in CP children, including muscle strength and spasticity. Most of the studies performed anodal stimulation on the injured side of the M1 area, the usual stimulation intensity was 1.0 mA, and each stimulation lasted 20 min (13–15).

However, for the stimulation dose, some researchers have different ideas. Among them, a study on CP children with unilateral hemiplegia selected a stimulus intensity of 1.5 mA for 20 min (16). The results showed that anode tDCS stimulation of the M1 area on the damaged side had immediate and short-term effects on hand functional flexibility. Another study selected a stimulation intensity of 0.7 mA for children with unilateral cerebral palsy, which confirmed its effect on exciting the corticospinal tract (12). Smoser et al. (17). applied direct current stimulation to the M1 area with an intensity of 1 mA for 15 min for 10 days and found that the stiffness and spasticity of the biceps brachii and extensor carpi radialis in CP children were significantly improved. This intervention duration was shorter than the duration of 20 min used in most studies, and an improvement effect was observed.

tDCS has a good application prospect for improving upper limb motor function in CP children. However, whether an optimal stimulation intensity and optimal intervention duration exist for improvement of upper limb function still requires further validation by clinical studies.

### Lower limb function

Few studies of the effect of tDCS on the lower extremity function of CP children are available, and the existing research mainly focuses on gait function and balance. In these studies, the main stimulation locations were the M1 area or the cerebellum (18–20). For CP children, anode tDCS stimulation of the M1 area or the cerebellum can help with gait patterns or balance to some extent, but the mechanism of action is not yet clear.

In an RCT study on the balance ability of CP children (21), VR combined with a synchronous tDCS intervention was performed on the children. The tDCS stimulation parameters were a 1-mA current lasting for 20 min with the anode placed on the M1 area and the cathode placed on the contralateral supraorbital region. The results showed that CP children who received true tDCS improved more than the control group children. The results from the 1-month follow-up demonstrated persistent improvements, which the researchers believe are related to the regulatory and remodeling effects of tDCS on the cerebral cortex. In addition, two case reports of tDCS interventions in CP children showed improved gait or balance function. Durate et al. (18) placed the anode at C4 and the cathode at C3 and performed stimulaton at 1 mA for 20 min for 10 sessions. The patient's gait score and children's balance ability scale scores were significantly improved. Santos (12) performed treadmill training combined with tDCS in a child with spastic cerebral palsy. They placed the anode electrode on the cerebellum area and the cathode electrode on the supraorbital crest and completed 5 sessions of tDCS intervention with a 1-mA current and 20 min of stimulation for each session. The results showed that the children's cadence, average speed and pelvic symmetry were improved.

No high-quality research is currently available to verify that the mechanism of tDCS interfering with balance function and gait patterns in CP children is consistent with some researchers' assumptions and whether modulation of the cortex can improve brain network connections in CP children and achieve brain function remodeling. However, related studies have found that tDCS increases activation of the prefrontal cortex during walking in Parkinson's disease patients and improves gait through modulation of the cortex and through the neural circuit. In future research, the mechanism of tDCS intervention on balance function and gait patterns in CP children warrants exploration to achieve precise modulation of motor function in CP children.

## tDCS improves cognitive and language functions in CP children

At present, only three reports are available on the application of tDCS to improve cognitive and language functions in CP children, two of which are case reports. Although the sample size involved is small, the results also demonstrate that tDCS can improve cognitive function and language and speech functions in CP children.

Ko et al. (22) divided CP children into an experimental group and a control group, with true tDCS combined with cognitive training in the experimental group and sham tDCS combined with cognitive training in the control group. The anode electrode was placed on the dorsal prefrontal cortex (DLPFC) of the more affected side, and the cathode electrode was placed on the contralateral supraorbital region. The results showed that the control group had a certain improvement in operation ability, attention transfer and comprehension ability, while tDCS combined with cognitive training significantly improved each assessed item, and the improvement degree in the experimental group was better than that in the control group. Lima et al. (23)reported a case of tDCS improving speech function. In this study, a child with cerebral palsy and speech apraxia was treated by tDCS 5 times a week for 2 weeks. During treatment, the anode electrode was placed in Broca's area, and the cathode electrode was placed in the contralateral supraorbital region; at the same time, oral movement and speech training were performed. After treatment, the child's oral performance, especially the flexibility of the tongue, was significantly improved. In addition, language fluency and consonant pronunciation were also improved. Lima et al. (24) published another report of tDCS treatment in a child with cerebral palsy in 2021; the child underwent two phases of tDCS combined with speech therapy for 10 sessions each. Anodal stimulation of Broca's area was performed in the first stage, and anodal stimulation of the left dorsolateral prefrontal cortex was performed in the second stage. After the second intervention stage, the children's vowel and consonant pronunciation and vocabulary were significantly improved compared to their abilities in the first stage. In addition, neuroimaging results showed myelin formation, which might reflect the mechanism of tDCS.

Few studies have focused on the application of tDCS to cognitive, language and speech functions in CP children. Therefore, further clinical research is needed for regional selection of tDCS interventions for cognitive and language functions in CP children. Meanwhile, many reports are available on the use of tDCS to improve cognitive and speech function in children with attention deficit hyperactivity disorder (ADHD) and autism spectrum disorder (ASD). Evidence from ADHD patients demonstrates that anode stimulation of the left dorsal prefrontal cortex (LDLPFC) can improve selective attention, executive control, and even walking ability (25, 26). Studies on ASD patients showed improvements in cognitive and language performance (27, 28). Consequently, we can speculate that the left dorsolateral prefrontal cortex, as a brain area closely related to cognition and language, can also play an important role in tDCS treatment for cognitive and language abilities in CP children.

## tDCS application prospects for other comorbidities in CP children

### Sleep disorder

More studies have explored the sleep quality of CP children (29, 30), and CP children are at risk of sleep disorders. Common problems include difficulty falling asleep, frequent waking at night, sleep-related respiratory disorders, early awakening and daytime fatigue. Sleep problems have been demonstrated to be related to the independence, quality of life and mental health of family members of CP children (31, 32). Another study found that sleep quality can affect the effect of speech therapy to some extent (33). Therefore, identifying and improving sleep problems as soon as possible are of great significance for CP children. However, tDCS has not been reported to improve sleep disorders in CP children.

However, tDCS has been widely used to treat sleep disorders in other populations and has been demonstrated to have a regulatory effect on sleep disorders (34, 35). A study of anodic tDCS stimulation in the LDLPFC of children with ASD found that the sleep questionnaire scores of children with ASD were significantly improved (36), and another tDCS intervention for student athletes also verified the increase in sleep time and subjective sleep improvement (37). At present, there is relevant evidence that the sleep of normal infants begins to resemble the typical adult state between the ages of 2 and 5 (38). According to various sleep related studies and DSM-5 standards (39), we can find that the symptoms of sleep disorders in CP children are similar to those in other patients with sleep disorders. Although there is no relevant study on tDCS intervening sleep disorder in children with CP, we can speculate that tDCS may be used to improve sleep disorder in children with CP over 5 years old according to the rules of children's sleep development and the similarity of sleep disorder symptoms, but the target and intensity of stimulation need to be verified.

### Anxiety

A study investigated the prevalence of comorbid anxiety disorder in CP children. Through evaluation of scale scores reported by parents and scale scores reported by children, 38 and 46% of CP children were found to have clinical anxiety symptoms, respectively, and some parents did not report the anxiety symptoms of their child even though the child had such symptoms (40), which confirms that anxiety symptoms are more prominent comorbid symptoms in CP children. On the other hand, the findings also suggest that parents might have ignored the anxiety symptoms of CP children. Anxiety symptoms in children may lead to fear, pain and other physical symptoms, which are not conducive to their social communication and may affect their quality of daily life and social participation (41).

tDCS has been demonstrated to have a regulatory effect on anxiety symptoms. It can effectively improve anxiety symptoms in college students, women with primary dysmenorrhea and elderly individuals (42–44). Patients with anxiety disorder have been shown to have imbalance in the activities of left and right DLPFC (45–47). One related literatures mention that the emotional and cognitive problems of CP children are closely related to the dysfunction in the frontoparietal network (48). As a technology that can modulate the activities of cortex, tDCS can balance the bilateral brain and modulate the frontoparietal network with dysfunction. Therefore, based on the clinical application related to anxiety and the similarity of frontoparietal network between children with CP and patients with anxiety disorder, we can assume that tDCS may also help improve the anxiety symptoms of CP children. In future applications, we can select the reasonable stimulation location and intensity according to existing research on tDCS interventions for anxiety symptoms and explore the effect of tDCS on the anxiety symptoms of CP children, which may help them.

### Safety of tDCS in children

Currently, the safety and tolerability data of tDCS in adults are relatively mature, and no serious adverse reactions have been reported. The main side effects reported include skin itching and stinging or burning sensations.

Zewdie et al. (49) conducted a study on the safety and tolerability of tDCS in children, and the most adverse reactions reported after the intervention were itching and burning, followed by tingling and headache. Only a few subjects experienced dizziness or nausea. In all subjects, no seizures were observed. In the study of Raess et al. (14), 1 child reported epilepsy. After evaluation by the pediatrician, the child was believed to have had focal epilepsy before receiving the tDCS intervention, and therefore, the epilepsy was determined to not be associated with the tDCS intervention. In addition, every subject in this experiment reported pruritus, similar to the findings of Zewdie et al. (49).

Although tDCS has been widely used in clinical practice and extensive evidence supports the safety and tolerability of tDCS in children, its foundation is still electrical stimulation therapy, implying that children with contraindications to electrical stimulation therapy should not be treated with tDCS. In addition, due to the particularities of children, the primary concern in clinical practice is still the safety of children. The stimulation parameters, stimulation intensity, stimulation time, and frequency of the intervention should be carefully considered and designed. However, because of children's poor cognitive and expressive abilities, they may not be able to provide timely and accurate feedback on treatment effects. The therapist must focus on the children's subjective feelings at all times and regularly confirm the children's skin condition, vital signs and other objective conditions to avoid adverse reactions during tDCS treatment.

## Conclusion

Although few studies on tDCS application in CP children are available, most research supports the effectiveness of tDCS on motor function, language and cognitive function in CP children. In these studies (19, 23, 50), stimulation locations, stimulation intensities, and intervention durations and frequencies differed. Whether optimal values exist for these specific parameters is not known and warrants further study.

In some studies, synchronized tDCS interventions and rehabilitation training were performed. This training mode shortens the intervention time for children, but whether this synchronized mode is better than other treatment modes and whether different sequences will produce different effects still require confirmation by large-scale studies. In addition, tDCS has good application prospects for other comorbid symptoms in CP children, such as sleep disorders and anxiety disorders. Overall, tDCS has good application prospects as an auxiliary means for the rehabilitation of CP children, although its mechanism of action requires further exploration.

## Author contributions

LT and YW conceptualized and designed the study and drafted the initial manuscript. JM, YL, and LW reviewed and revised the manuscript. CS supervised the whole procedure. All authors contributed to the article and approved the submitted version.

## Funding

This work was supported by the National Key Research and Development Program of China (Grant No. 2018YFC2001600/04), the National Natural Science Fund (Grant No. 81874035), and the Clinical Science and Technology Innovation Project of Shanghai Shen Kang Hospital Development Center (Grant No. SHDC12018126).

## Conflict of interest

The authors declare that the research was conducted in the absence of any commercial or financial relationships that could be construed as a potential conflict of interest.

## Publisher's note

All claims expressed in this article are solely those of the authors and do not necessarily represent those of their affiliated organizations, or those of the publisher, the editors and the reviewers. Any product that may be evaluated in this article, or claim that may be made by its manufacturer, is not guaranteed or endorsed by the publisher.

## References

[1] Sadowska M Sarecka-Hujar B Kopyta I. Cerebral palsy: current opinions on definition, epidemiology, risk factors, classification and treatment options. Neuropsychiatr Dis Treat. (2020) 16:1505–18. 10.2147/NDT.S235165 32606703 PMC7297454

[2] Bugler KE Gaston MS Robb JE. Distribution and motor ability of children with cerebral palsy in Scotland: a registry analysis. Scott Med J. (2019) 64:16–21. 10.1177/0036933018805897 30336740

[3] Fluss J Lidzba K. Cognitive and academic profiles in children with cerebral palsy: a narrative review. Ann Phys Rehabil Med. (2020) 63:447–56. 10.1016/j.rehab.2020.01.005 32087307

[4] Yang S Xia J Gao J Wang L. Increasing prevalence of cerebral palsy among children and adolescents in China 1988–2020: a systematic review and meta-analysis. J Rehabil Med. (2021) 53. 10.2340/16501977-2841 33961057 PMC8814846

[5] Kuliński W Zukowska M. Cerebral palsy: clinical and social problems. Wiadomosci Lek Wars Pol 1960. (2019) 72:2261–68. 10.36740/WLek201912101 32124738

[6] Shah-Basak PP Wurzman R Purcell JB Gervits F Hamilton R. Fields or flows? A comparative metaanalysis of transcranial magnetic and direct current stimulation to treat post-stroke aphasia. Restor Neurol Neurosci. (2016) 34:537–58. 10.3233/RNN-150616 27163249

[7] Altamura CA. Transcranial direct current stimulation for autistic disorder. Biol Psychiatry. (2014) 76:e5–6. 10.1016/j.biopsych.2013.11.009 24342925

[8] Stagg CJ Nitsche MA. Physiological basis of transcranial direct current stimulation. Neuroscientist. (2011) 17:37–53. 10.1177/1073858410386614 21343407

[9] Wimalasundera N Stevenson VL. Cerebral palsy. Pract Neurol. (2016) 16:184–94. 10.1136/practneurol-2015-001184 26837375

[10] Ciechanski P Kirton A. Transcranial direct-current stimulation can enhance motor learning in children. Cereb Cortex. (2017) 27:2758–67. 10.1093/cercor/bhw114 27166171

[11] Chen CY Rich T Cassidy J Gillick B. Corticospinal excitability in children with congenital hemiparesis. Brain Sci. (2016) 6:49. 10.3390/brainsci6040049 27775599 PMC5187563

[12] Nemanich ST Rich TL Chen CY Menk J Rudser K Chen M. Influence of combined transcranial direct current stimulation and motor training on corticospinal excitability in children with unilateral cerebral palsy. Front Hum Neurosci. (2019) 13:137. 10.3389/fnhum.2019.00137 31105541 PMC6492624

[13] Feng W Kautz SA Schlaug G Meinzer C George MS Chhatbar PY. Transcranial direct current stimulation for poststroke motor recovery: challenges and opportunities. PMR. (2018) 10:S157–64. 10.1016/j.pmrj.2018.04.012 30269802 PMC7153501

[14] Raess L Hawe R Metzler M Zewdie E Condliffe E Dukelow S. A robotic rehabilitation and transcranial direct current stimulation in children with bilateral cerebral palsy. Front Rehabil Sci. (2022) 3:843767. 10.3389/fresc.2022.843767 36188922 PMC9397997

[15] Friel KM Lee P Soles LV Smorenburg ARP Kuo HC Gupta D. Combined transcranial direct current stimulation and robotic upper limb therapy improves upper limb function in an adult with cerebral palsy. Neuro Rehabilitation. (2017) 41:41–50. 10.3233/NRE-171455 28505986 PMC5546204

[16] Inguaggiato E Bolognini N Fiori S Cioni G. Transcranial direct current stimulation (tDCS) in unilateral cerebral palsy: a pilot study of motor effect. Neural Plast. (2019) 2019:1–10. 10.1155/2019/2184398 30733800 PMC6348802

[17] Smoter M Jedrzejczyk-Góral B Chen A Ciszek B Ignasiak Z. Effects of tDCS on muscle stiffness in children with cerebral palsy measured by myotonometry: a preliminary study. Appl Sci. (2020) 10:2616. 10.3390/app10072616

[18] Duarte N Grecco L Lazzari R Galli M Oliveira C. P082—Effect of bilateral tDCS on functional balance and the Gait profile score in a child with hemiparetic spastic cerebral palsy. Gait Posture. (2018) 65:365–6. 10.1016/j.gaitpost.2018.07.017

[19] Santos LV Gaspar BC Zacarias LR de Souza TF Lopes JBP Duarte NAC. Cerebellar tDCS during treadmill training for a child with dystonic cerebral palsy: a case report. Gait Posture. (2019) 73:288–9. 10.1016/j.gaitpost.2019.07.272

[20] Grecco LAC Oliveira CS Duarte N de AC Lima VLCC Zanon N Fregni F. Cerebellar transcranial direct current stimulation in children with ataxic cerebral palsy: a sham-controlled, crossover, pilot study. Dev Neurorehabilitation. (2017) 20:142–8. 10.3109/17518423.2016.1139639 27003795

[21] Lazzari RD Politti F Belina SF Collange Grecco LA Santos CA Dumont AJL. Effect of transcranial direct current stimulation combined with virtual reality training on balance in children with cerebral palsy: A randomized, controlled, double-blind, clinical trial. J Mot Behav. (2017) 49:329–36. 10.1080/00222895.2016.1204266 27644454

[22] Ko EJ Hong MJ Choi EJ Yuk JS Yum MS Sung IY. Effect of anodal transcranial direct current stimulation combined with cognitive training for improving cognition and language among children with cerebral palsy with cognitive impairment: a pilot, randomized, controlled, double-blind, and clinical trial. Front Pediatr. (2021) 9:713792. 10.3389/fped.2021.713792 34513765 PMC8424100

[23] Carvalho Lima VLC Collange Grecco LA Marques VC Fregni F Brandão de. Ávila CR. Transcranial direct current stimulation combined with integrative speech therapy in a child with cerebral palsy: a case report. J Bodyw Mov Ther. (2016) 20:252–7. 10.1016/j.jbmt.2015.03.007 27210840

[24] Lima VLCC Cosmo C Lima KB Martins MA Rossi SG Collange Grecco LA. Neuromodulation: a combined-therapy protocol for speech rehabilitation in a child with cerebral palsy. J Bodyw Mov Ther. (2022) 29:10–15. 10.1016/j.jbmt.2021.09.002 35248256

[25] Bandeira ID Guimarães RS Jagersbacher JG Barretto TL de Jesus-Silva JR Santos SN. Transcranial direct current stimulation in children and adolescents with attention-deficit/hyperactivity disorder (ADHD): a pilot study. J Child Neurol. (2016) 31:918–24. 10.1177/0883073816630083 26879095

[26] Soltaninejad Z Nejati V Ekhtiari H. Effect of anodal and cathodal transcranial direct current stimulation on DLPFC on modulation of inhibitory control in ADHD. J Atten Disord. (2019) 23:325–32. 10.1177/1087054715618792 26689935

[27] Amatachaya A Auvichayapat N Patjanasoontorn N Suphakunpinyo C Ngernyam N Aree-Uea B. Effect of anodal transcranial direct current stimulation on autism: a randomized double-blind crossover trial. Behav Neurol. (2014) 2014:1–7. 10.1155/2014/173073 25530675 PMC4230001

[28] Schneider HD Hopp JP. The use of the bilingual aphasia test for assessment and transcranial direct current stimulation to modulate language acquisition in minimally verbal children with autism. Clin Linguist Phon. (2011) 25:640–54. 10.3109/02699206.2011.570852 21631313

[29] Löwing K Gyllensvärd M Tedroff K. Exploring sleep problems in young children with cerebral palsy—a population-based study. Eur J Paediatr Neurol. (2020) 28:186–92. 10.1016/j.ejpn.2020.06.006 32669213

[30] Sandella DE O'Brien LM Shank LK Warschausky SA. Sleep and quality of life in children with cerebral palsy. Sleep Med. (2011) 12:252–6. 10.1016/j.sleep.2010.07.019 21334973 PMC3065038

[31] Ghorbanpour Z Hosseini SA Akbarfahimi N Rahgozar M. Correlation between sleep disorders and function in children with spastic cerebral palsy. Iran J Child Neurol. (2019) 13:35–44. 31327967 PMC6586452

[32] Lélis AL Cardoso MV Hall WA. Sleep disorders in children with cerebral palsy: an integrative review. Sleep Med Rev. (2015) 30:63–71. 10.1016/j.smrv.2015.11.008 26874066

[33] Herrmann O Ficek B Webster KT Frangakis C Spira AP Tsapkini K. Sleep as a predictor of tDCS and language therapy outcomes. Sleep. (2022) 45:zsab275. 10.1093/sleep/zsab275 34875098 PMC8919198

[34] Frase L Piosczyk H Zittel S Jahn F Selhausen P Krone L. Modulation of total sleep time by transcranial direct current stimulation (tDCS). Neuropsychopharmacology. (2016) 41:2577–86. 10.1038/npp.2016.65 27143601 PMC4987856

[35] Frase L Jahn F Tsodor S Krone L Selhausen P Feige B. Offline bi-frontal anodal transcranial direct current stimulation decreases total sleep time without disturbing overnight memory consolidation. Neuromodulat Technol Neural Interface. (2021) 24:910–5. 10.1111/ner.13163 32394544

[36] Qiu J Kong X Li J Yang J Huang Y Huang M. Transcranial direct current stimulation (tDCS) over the left dorsal lateral prefrontal cortex in children with autism spectrum disorder (ASD). Neural Plast. (2021) 2021:1–11. 10.1155/2021/6627507 34257640 PMC8245257

[37] Charest J Marois A Bastien CH. Can a tDCS treatment enhance subjective and objective sleep among student-athletes? J Am Coll Health. (2021) 69:378–89. 10.1080/07448481.2019.1679152 31724914

[38] Karthikeyan R Cardinali DP Shakunthala V Spence DW Brown GM Pandi-Perumal SR. Understanding the role of sleep and its disturbances in Autism spectrum disorder. Int J Neurosci. (2020) 130:1033–46. 10.1080/00207454.2019.1711377 31903819

[39] van Bemmel AL Kerkhof GA. Sleep-wake disorders and DSM-5. Tijdschr Psychiatr. (2014) 56:192–5. 24643830

[40] McMahon J Harvey A Reid SM May T Antolovich G. Anxiety in children and adolescents with cerebral palsy. J Paediatr Child Health. (2020) 56:1194–200. 10.1111/jpc.14879 32412671

[41] Sackl-Pammer P Özlü-Erkilic Z Jahn R Karwautz A Pollak E Ohmann S. Somatic complaints in children and adolescents with social anxiety disorder. Neuropsychiatr Klin Diagn Ther Rehabil Organ Ges Osterreichischer Nervenarzte Psychiater. (2018) 32:187–95. 10.1007/s40211-018-0288-8 30218392 PMC6290697

[42] Garcia S Nalven M Ault A Eskenazi MA. tDCS as a treatment for anxiety and related cognitive deficits. Int J Psychophysiol. (2020) 158:172–7. 10.1016/j.ijpsycho.2020.10.006 33129848

[43] Dutra LRDV Pegado R Silva LK da Silva Dantas H Câmara HA Silva-Filho EM. Modulating anxiety and functional capacity with anodal tDCS over the left dorsolateral prefrontal cortex in primary dysmenorrhea. Int J Womens Health. (2020) 12:243–251. 10.2147/IJWH.S226501 32308497 PMC7147620

[44] Brooks H Oughli HA Kamel L Subramanian S Morgan G Blumberger DM. Enhancing cognition in older persons with depression or anxiety with a combination of mindfulness-based stress reduction (MBSR) and transcranial direct current stimulation (tDCS): results of a pilot randomized clinical trial. Mindfulness. (2021) 12:3047–59. 10.1007/s12671-021-01764-9 34630733 PMC8491443

[45] Grimm S Beck J Schuepbach D Hell D Boesiger P Bermpohl F. Imbalance between left and right dorsolateral prefrontal cortex in major depression is linked to negative emotional judgment: an fMRI study in severe major depressive disorder. Biol Psychiatry. (2008) 63:369–376. 10.1016/j.biopsych.2007.05.033 17888408

[46] Nitschke JB Heller W. Distinguishing neural substrates of heterogeneity among anxiety disorders. Int Rev Neurobiol. (2005) 67:1–42. 10.1016/S0074-7742(05)67001-8 16291018

[47] Stein DJ Fernandes Medeiros L Caumo W Torres IL. Transcranial direct current stimulation in patients with anxiety: current perspectives. Neuropsychiatr Dis Treat. (2020) 16:161–69. 10.2147/NDT.S195840 32021208 PMC6969693

[48] Qin Y Li Y Sun B He H Peng R Zhang T. Functional connectivity alterations in children with spastic and dyskinetic cerebral palsy. Neural Plast. (2018) 2018:7058953. 10.1155/2018/7058953 30186320 PMC6114065

[49] Zewdie E Ciechanski P Kuo HC Giuffre A Kahl C King R. Safety and tolerability of transcranial magnetic and direct current stimulation in children: prospective single center evidence from 3.5 million stimulations. Brain Stimulat. (2020) 13:565–75. 10.1016/j.brs.2019.12.025 32289678

[50] Auvichayapat P Aree-Uea B Auvichayapat N Phuttharak W Janyacharoen T Tunkamnerdthai O. Transient changes in brain metabolites after transcranial direct current stimulation in spastic cerebral palsy: a pilot study. Front Neurol. (2017) 8:366. 10.3389/fneur.2017.00366 28824525 PMC5534437

